# Correlation of Experimental and Calculated Inhibition Constants of Protease Inhibitor Complexes

**DOI:** 10.3390/ijms25042429

**Published:** 2024-02-19

**Authors:** Peter Goettig, Xingchen Chen, Jonathan M. Harris

**Affiliations:** 1 Department of Pharmaceutical and Medicinal Chemistry, Institute of Pharmacy, Paracelsus Medical University, Strubergasse 21, 5020 Salzburg, Austria; 2 Institute of Health and Biomedical Innovation, Queensland University of Technology, Brisbane, QLD 4059, Australia or chenxingchen@sibcb.ac.cn (X.C.); j2.harris@qut.edu.au (J.M.H.)

**Keywords:** dissociation constant, free energy, hirudin, inhibition constant, protease inhibitor, SARS-CoV-2 main protease, sunflower trypsin inhibitor, Van ’t Hoff equation

## Abstract

Predicting the potency of inhibitors is key to in silico screening of promising synthetic or natural compounds. Here we describe a predictive workflow that provides calculated inhibitory values, which concord well with empirical data. Calculations of the free interaction energy ΔG with the YASARA plugin FoldX were used to derive inhibition constants K_i_ from PDB coordinates of protease–inhibitor complexes. At the same time, corresponding K_D_ values were obtained from the PRODIGY server. These results correlated well with the experimental values, particularly for serine proteases. In addition, analyses were performed for inhibitory complexes of cysteine and aspartic proteases, as well as of metalloproteases, whereby the PRODIGY data appeared to be more consistent. Based on our analyses, we calculated theoretical K_i_ values for trypsin with sunflower trypsin inhibitor (SFTI-1) variants, which yielded the more rigid Pro14 variant, with probably higher potency than the wild-type inhibitor. Moreover, a hirudin variant with an Arg1 and Trp3 is a promising basis for novel thrombin inhibitors with high potency. Further examples from antibody interaction and a cancer-related effector-receptor system demonstrate that our approach is applicable to protein interaction studies beyond the protease field.

## 1. Introduction

There are numerous software packages and web servers that can be utilized to calculate the interaction energies and equilibrium constants of all biological molecule and ligand types [1,2,3]. For instance, the sophisticated screening for computer generated inhibitors of a galactofuranosyl-transferase highlights the connection between the free binding energy ΔG (Gibbs free energy) in the complex and the corresponding inhibition constant K_i_ [4]. Molecular docking and molecular dynamics, the three-dimensional quantitative structure-activity relationship (3D-QSAR) and in silico ADMETox allowed to calculate theoretical inhibition constants K_i_ according to the formula K = exp(ΔG/RT), as well as the expected phamacokinetic behavior. Basically, K is the equilibrium constant K_eq_ of a chemical reaction that can be an association constant K_a_ or a dissociation constant K_D_. By logarithmizing this formula the standard Van ’t Hoff equation ΔG = −RT•ln K is obtained, whereby either multiplication or division with the standard concentration mol/liter result in the required pure number for K [5]. In competitive inhibition a reversible inhibitor competes with the substrate for one binding site forming either ES or EI enzyme complexes, whereas in non-competitive, uncompetitive and mixed inhibition an enzyme–substrate–inhibitor complex (ESI) can be formed via a second binding site for reversible inhibitors [6,7]. Non-competitive inhibitors bind independent of the substrate, while uncompetitive inhibitors can only bind to the ES complex. In addition, such ESI complexes are often not available in terms of structural coordinates in contrast to enzyme-inhibitor complexes of competitive inhibition. Nevertheless, K_i_ values can be interpreted as dissociation constants K_D_ of competitively binding inhibitor-enzyme complexes, as demonstrated for the chymotrypsin C-ecotin system [8].

Since the beginning of the COVID-19 pandemic numerous computational studies focused on potential inhibitors of the SARS-CoV-2 main protease (MPro), a cysteine protease [9]. Also, inhibitors of the cancer-related matrix metalloproteinases (MMPs) are of high interest. Thus, structure based virtual screenings followed by in vitro assays have been undertaken [10]. An in silico prediction of the inhibitory constants K_i_ of compounds directed against thrombin, the central serine protease in blood coagulation, was performed by machine learning [11]. Previous studies have utilized molecular simulation platforms such as VMD (http://www.ks.uiuc.edu/Research/vmd/, accessed on 19 January 2024) and NAMD (http://www.ks.uiuc.edu/Research/namd/, accessed on 19 January 2024) to design highly potent peptidic inhibitors [12,13]. This approach involved serine protease as targets in molecular dynamics (MD) simulations of various inhibitor complexes, where different amino acid substitutions were made in the peptide sequence to maximize all molecular interactions with a focus on the hydrogen bonding network during the simulation [14,15]. Advanced program suites, such as the MOE suite offer various docking options and quantum mechanical calculations for drug discovery [16].

In our study, we attempted a relatively simple approach to assess free binding energies of polypetidic inhibitors and corresponding inhibition constants K_i_ with the YASARA program suite [17]. YASARA provides a wide range of graphical tools for protein modeling, molecular dynamics simulations and structural analysis including virtual reality options for various operating systems, such as Windows, Linux, Mac OS and Android. In particular, these calculations were conducted with the YASARA plugin FoldX, which allows to analyze protein stability, protein-protein interactions and protein-ligand binding affinities using empirical force fields [18,19]. Both programs can handle biomolecular assemblies of proteins, nucleic acids, carbohydrates, and lipids. The FoldX results could often be confirmed or surpassed by data from the web-server PRODIGY that predicts protein-protein and ligand binding affinities expressed as free binding energy and K_D_ values using machine learning algorithms (https://wenmr.science.uu.nl/prodigy/, accessed on 28 January 2024) [20,21]. In addition, about a dozen associated web services of the PRODIGY server can analyze protein interactions from potential docking sites to model fitting into electron densities.

As our approach is applicable to all classes of proteases, it can help more experimentally oriented laboratories to find potent polypetidic inhibitor mutants for their protease studies without time consuming MD calculations by specialist groups. Moreover, the free interaction energies and the calculated K_D_ values of other biomolecular systems may be valuable beyond the field of protease research.

## 2. Results and Discussion

**Figure 1:**
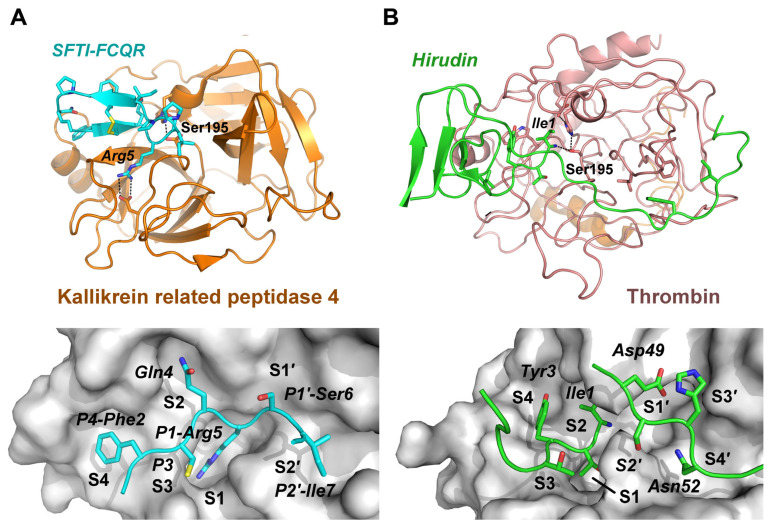
Exemplary complex structures of trypsin-like serine proteases. (**A**) KLK4 complex with a highly potent SFTI-1 variant (cyan), containing Arg5 instead of the natural Lys5, as well as the mutations Phe2, Gln4, and Asn14 (**upper panel**). The lower panel shows a close-up of the active site, in which the P4 to P2′ residues of the SFTI variant bind to the corresponding S4 to S2′ specificity pockets as other canonical inhibitors similar to substrates via the standard mechanism. (**B**) Human α-thrombin in complex with the extremely strong inhibitor hirudin (green), an anticoagulant from the leech *Hirudo medicinalis* (**upper panel**). In contrast to canonical inhibitors hirudin binds in a reverse manner, with the N-terminal Ile1 occupying the S2 subsite, Thr2 the S1 subsite, and Tyr3 the S4 subsite (**lower panel**). However, Asp49 to Asn52 of hirudin correspond to P1′ to P4′ residues and bind the S1′ to S4′ subsites like canonical inhibitors, whereby further protease–inhibitor interactions occur in the prime side.

The cyclic sunflower trypsin inhibitor (SFTI-1) with the sequence Gly1-Arg2-Cys3-Thr4-Lys5-Ser6-Ile7-Pro8-Pro9-Ile10-Cys11-Phe12-Pro13-Asp14 is the paragon of a highly specific inhibitor with engineered variants for several trypsin-like proteases, which has been used in numerous enzymatic and structural studies [22]. SFTI-1 inhibits the target protease through the standard mechanism, with its reactive loop binding to the protease active site in a substrate-like manner [23]. Its recognition sequence P4 to P2′ is ideally suited to bind the specificity pockets S4 to S2′ of the target protease according to the Schechter-Berger nomenclature (Figure 1A) [24]. The P1 residue Lys5 acts as the key specificity determinant by binding to the S1 subsite of the target protease, while the residues Thr4 and Arg2 interact with the S2 and S4 subsites of the target protease, respectively. Three proline residues, the disulfide Cys3-Cys11, and the short internal β-sheet render the scaffold of SFTI-1 very rigid, which is thought to contribute to the strong potency of this inhibitor. In addition, SFTI-1 can be easily engineered by single and multiple mutations in order to increase its potency with respect to target proteases, such as coagulating factors, plasmin, kallikrein-related peptidases and others [14]. For example the SFTI variant GFCQRSIPPICFPN was an excellent inhibitor of human kallikrein-related peptidase 4 (KLK4) with a picomolar K_i_ and its X-ray structure was determined to high resolution (Figure 1A, Table 1). Otherwise, several natural inhibitors of proteases, such as trypsin with bovine pancreatic trypsin inhibitor (BPTI) and blood coagulation factor II, thrombin, with hirudin are inhibited in the femtomolar range (Figure 1B, Table 1).

**Figure 2:**
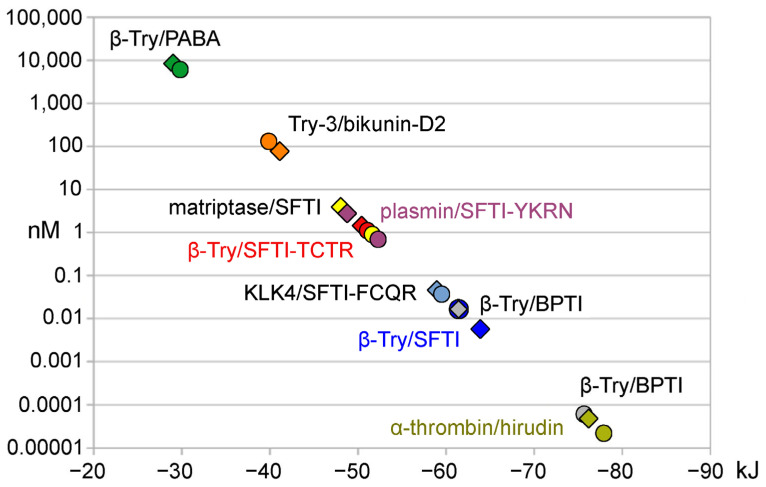
Plot of K_i_ values (nM) in logarithmic scale versus ΔG (kJ) for serine protease inhibitor complexes. The round symbols represent experimental K_i_ and ΔG values from protease–inhibitor pairs, while the diamonds belong to calculated K_i_ (K_D_) and ΔG derived from protease inhibitor complex coordinates: β-Try/PABA, Try-3/bikunin-D2, matriptase-SFTI, plasmin-SFTI, β-Try/SFTI-TCTR-N12-N14, KLK4/SFTI-FCQR-N14, β-Try/BPTI, and α-thrombin/hirudin (more β-Try structures with SFTI-1 variants are available). Essentially, free interaction energies were calculated with the YASARA plugin FoldX or with the web server PRODIGY. Overall, the FoldX results for serine protease inhibitor complexes correlated better with the experimental data. More details can be found in Table 1.

**Figure 3:**
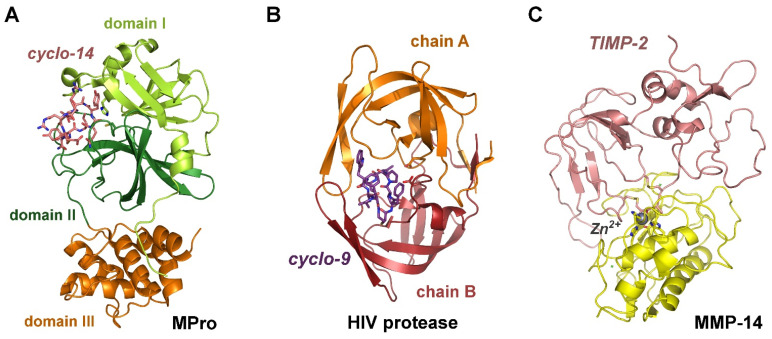
Examples of cysteine, aspartic and metalloproteases. (**A**) SARS-CoV-2 MPro is a chymotrypsin-like protease with a catalytic dyad (His41, Cys145) in the half domains I and II, while domain III mediates dimerization (PDB 7RNW). The synthetic, cyclo-14-mer inhibits with a K_i_ of roughly 4 nM. (**B**) Aspartic HIV protease forms a symmetrical active dimer, which binds a synthetic cyclo9-mer exhibiting an estimated K_i_ of 3 nM (PDB 7YF6). (**C**) The catalytic domain of MMP-14 (MT1-MMP) binds the natural proteinaceous inhibitor TIMP-2 via a tight interaction to Zn^2+^ from the N-terminal Cys1 and Thr2 in the S1′ pocket (PDB 1BUV), exhibiting a K_i_ of 104 pM.

Overall, our approach works very well for protease–inhibitor complexes, which consist of serine proteases and polypeptidic inhibitors (Figure 2). The only exception was the trypsin-BPTI complex, which was reported to have a covalent nature, and thus exhibited poor correlation of calculated and measurement based free interaction energy ΔG. Nevertheless, minor modifications are tolerated in these calculations as in the acetyl group containing cyclic 14-mer inhibitor of SARS-CoV-2 MPro, a chymotrypsin-like cysteine protease (Figure 3A) [40]. In addition, the N-methylation of Phe1 and the β-thio-ε-amino acid linker in the cyclic 9-mer inhibitor of the aspartic HIV protease result in consistent free binding energies (Figure 3B) [42]. A tentative calculation with PRODIGY for the energy minimized BACE-1/22-mer polypeptide complex improved the correlation with the experimental values to some extent. Thus, future studies with respect to the prediction for mutant protease and inhibitor interactions might benefit from such thorough preparation and modification of the coordinate files.

**Figure 4:**
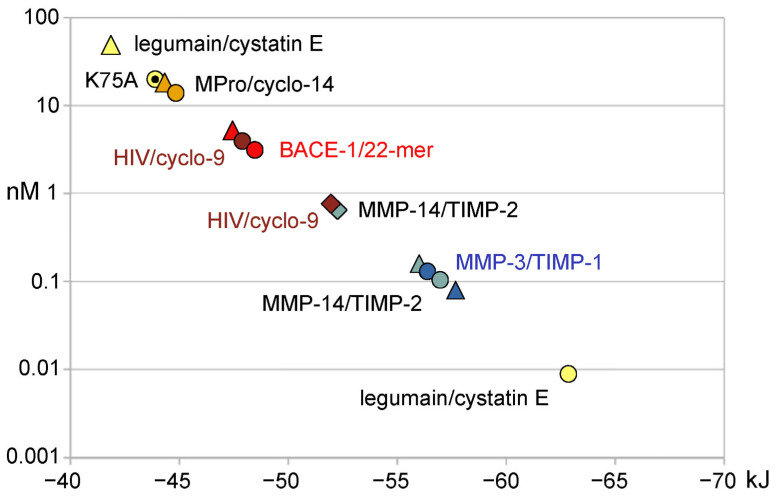
Plot of K_i_ values (nM) in logarithmic scale versus ΔG (kJ) for cysteine, aspartic and metallo-protease inhibitor complexes. The round symbols represent experimental K_i_ and ΔG values from protease–inhibitor pairs, while the diamonds and triangles belong to calculated K_i_ (K_D_) and ΔG derived from calculations with the YASARA plugin FoldX and the PRODIGY web server, respectively. The protease inhibitor complexes were legumain/cystatin E, SARS-CoV-2 Mpro/cyclo-14-mer, BACE-1/22-mer, HIV protease/cyclo-9-mer, MMP-14/TIMP-2, and MMP-3/TIMP-1. In five cases the correlation of experimental data was better with PRODIGY results. The cystatin E-K75A constant (19.8 nM) for human legumain corresponds better to the one derived from the coordinates of the structural data (46.4 kJ/mol) compared with the reported 0.011 nM. A better correlation was seen for energy minimized coordinates of the BACE-1 complex (−47.28 kJ/mol). More details can be found in Table 1.

It has to be mentioned that occasionally the calculations of FoldX and the PRODIGY server resulted in completely discrepant or inconsistent values of the free Gibbs interaction energy compared to the experimentally derived data (Table 1, Figure 4). For example, this phenomenon was observed for human legumain (AEP) in complex with human cystatin E, which was reported to have an inhibition constant of about 11 pM, while the calculated K_i_ was 46.4 nM (Table 1) [38]. These experimental data were measured using human cystatin E and glycosylated legumain, which may have shifted the K_i_ to some extent. Interestingly, the cystatin E-K75A mutant exhibited a K_i_ of 19.8 nM with human legumain, which is much closer to the calculated data of ΔG and K_i_ from the PDB 4N6O [39,47]. All results from simulation attempts with or without energy minimization and even deletion of the N-glycans of legumain did not come near the reported experimental picomolar K_i_ (Figure 4). The presence of succinimide (SNN) converted from Asp147 might have an impact on the calculated values.

In case of the metalloprotease complexes of MMP-3/TIMP-1 and MMP-14/TIMP-2 with a Zn^2+^ in the catalytic center, some erratic data were obtained with FoldX until the metal ion LINK records in the PDB were removed. Whereas the PRODIGY server yielded exactly the same results with and without the catalytic Zn^2+^, which were consistent with the experimental K_i_ values (Figure 3C) [43,45]. Altogether the performance of the PRODIGY server was better for the cysteine and aspartic protease examples, as well as for the two metalloprotease complexes (Figure 4). Larger discrepancies of experimental and calculated K_i_ and ΔG values may arise from differences in the protein and polypeptide molecules employed in enzyme kinetic assays and crystallization procedures. However, crystallization artifacts, such as the presence of precipitants and the frozen state of measurements with a temperature of 100 K, should be largely eliminated by removing most HETATM entries from the PDB and by energy minimization. Nevertheless, the crystal structure coordinates may still contain significant differences with respect to the molecular polypeptide structures in solution, in particular, more flexible and alternative conformations of loops and side-chains. Similar procedures are performed with KiDoQ for virtual screening and scoring of inhibitory compounds with the AutoDock4 suite, which calculated inhibition constants K_i_ from QSAR energy terms, followed by comparing the theoretical K_i_ values to experimentally available ones [48]. A correlation function allowed for further predictions, whereby a three energy-based descriptor based QSAR approach performed better than an SVM model with six descriptors. As the FoldX plugin of YASARA was developed for polypeptides, the calculation of free binding energies for modeled inhibitor complexes could serve as a simple and straightforward tool to assess potency changes for mutations of residues at the protease–inhibitor interface. Unfortunately, the more advanced program suites AMBER22 or 23, CHARMM, and GROMACS 2023 (https://doi.org/10.5281/zenodo.10017699) are not so easy to install and to start on LINUX, Mac OS or Windows systems [49,50,51]. Nevertheless, YASARA and FoldX or the PRODIGY server are recommended for experiment oriented research groups that do not specialize in molecular dynamics computing. A drawback of the PRODIGY server might be flawed calculations due to anisotropic B-factors or alternate conformations, while the option to include synthetic ligands in PRODIGY-LIGAND is advantageous. Nevertheless, it is possible to remove such unwanted factors in the graphical software COOT v0.9.8.92 and with PDBCUR (e.g., the mostprob option) of the CCP4 program suite [52,53]. Altogether, our approach is based on the premise that the lowest achievable energy of the complex in silico represents the “real” state, while no multiple conformations were considered, which are obtained in various runs of extended molecular dynamics simulations. Moreover, the experimental K_i_ values sample potential molecular conformations, which may depend on either the conformational selection or the induced fit mechanism, resulting in an averaged value [54]. Automated processing of in silico mutations may significantly speed up the computing time for screening potentially useful protease mutants and their polypetidic inhibitors. 

In order to outline a strategy for using both FoldX and the PRODIGY server as prediction tools, we attempted the following. Starting with the SFTI-TCTR variant encompassing the full sequence GTCTRSIPPICNPN with a K_i_ of 0.70 nM [15]. Interestingly, this variant inhibited the chymotryptic kallikrein-related peptidase KLK7 with a K_i_ of 17 nM. A systematic series of Ala mutants served as a guideline to reach or surpass the inhibition constant of the natural SFTI-1, GRCTKSIPPICFPD [32]. In this study a K_i_ of 0.017 was reported for the inhibition of β-trypsin, in very good concordance with the value of 0.007 nM derived from the FoldX calculation (Table 2). 

The coordinate files SFI1 and 6BVH were modified in COOT and then subjected to the abovementioned procedure in YASARA, before running both the FoldX and PRODIGY calculations. Interestingly, the calculated K_i_ for the SFTI-R5K variant equals that of SFTI-1. Apparently, the variant SFTI-TCTR-N12P14 was the best β-trypsin inhibitor in the series of PRODIGY calculations, which can be explained by its increased overall rigidity. 

The highly potent hirudin inhibitor of the blood coagulation factor thrombin has pharmacological significance, since more stable recombinant variants are applied as antithrombotic drugs [55]. Engineered hirudins with a Phe or Trp in position 3 enhance the binding affinity to thrombin up to 6-fold [56]. Both hirudin variants bind in the reverse mode with Val1/Ile1 and Tyr3 occupying the S1 and S4 subsites of thrombin [37]. Similar to the procedure for SFTI-1 variants, our calculations for an Arg1 residue as enhancer of the binding affinity may support improvement of the currently known antithrombotics.

**Table 2:** Inhibition constants K_i_ for β-trypsin and SFTI-1 (PDB 1SFI), SFTI-TCTR (PDB 6BVH) and in silico variants calculated with FoldX and the PRODIGY server. For comparison the available experimental data are specified. The coordinates were processed and energy minimized as described. The thrombin-hirudin complexes exhibit femtomolar K_D_ values. Both the hirudin 1 and 2 variants are reverse binding inhibitors with the sequences Val1-Val2-Tyr3 and Ile1-Thr2-Tyr3. Recombinant forms of hirudin variant 1 were employed by Lazar and coworkers [56].

β-Trypsin/SFTI-1	Measured K_i_ [nM]	FoldX K_i_ [nM]	PRODIGY K_D_ [nM]
SFTI-1	0.017	0.007	0.963
SFTI-P14	-	0.766	1.4
SFTI-R5	0.027	-	0.99
SFTI-TCTR-N12N14	0.7	2.86	-
SFTI-RCTR			4.7
SFTI-RCTK			9.9
SFTI-TCTK			5.5
SFTI-TCTK-P14			1.6
SFTI-TCTR-P14			0.43
**Thrombin/hirudin**	**Measured K_i_ [fM]**	**FoldX K_i_ [fM]**	**PRODIGY K_D_ [fM]**
hirudin-v1/v2	22	19	-
rhir-v1	180	-	-
rhir-v1-Trp3	60	-	-
rhir-v1-Phe3	30	-	-
hirudin-v2		15	-
v2-Trp3		0.077	-
v2-Arg1-Trp3		0.021	-

In principle, this strategy can be employed to assess the function of proteases and their interaction with substrates and inhibitors as well as for corresponding interactions of polypeptidic biological and synthetic systems. A study of the antibody fragment-nanobody complex Fab19-TC-Nb4 reported a K_D_ of 860 pM, whereby the FoldX analysis of chains “A, B” (Fab) and “a” (Nb) of the cryo-EM derived PDB 7RTH resulted in a calculated K_D_ of 250 pM, whereas the result of the PRODIGY server was in the higher nanomolar range [57]. Another example is the human colorectal cancer-related regulator protein adenomatous polyposis coli (APC) and its receptor Asef, which can be inhibited by peptidomimetics [58]. The nonapeptide MAI-150 exhibits a K_i_ of 120 nM and has a K_D_ of 250 nM in isothermal titration calorimetry. FoldX and the PRODIGY server yielded calculated values for the PDB 5IZ6 of 670 nM and 9 nM, respectively, which demonstrates the potential of our simple strategy for biological systems beyond protease–inhibitor complexes and could be adapted for screening in silico inhibitor libraries.

## 3. Material and Methods

In our standard procedure, we deleted all HETATM entries from the respective PDB files, whereby water could be kept, loaded the modified coordinates into YASARA and added hydrogen atoms using the CLEAN option. Then the AMBER99 force field was chosen, the simulation was initiated at 298 K and the pH defined according to the inhibition assays, followed by filling a simulation cell with water molecules and Na^+^ and Cl^−^ ions for neutralization under density control. After the solvent molecular dynamics (MD) had finished, an energy minimization for protein chains and all other molecules was run. Then the FoldX plugin was initialized and the interaction energy was calculated for the protease and the bound inhibitor, which yielded ΔG values in kcal/mol that were converted into the standard SI unit kJ/mol. For calculations with the PRODIGY web-server, original PDB coordinate files without modifications were uploaded to the interface with the URL https://wenmr.science.uu.nl/prodigy/ (accessed on 28 January 2024). Optionally, PDB coordinates were employed that had been subjected to the aformententioned energy minimization procedure in YASARA.

## Figures and Tables

**Table 1:** Interaction energies as Gibbs free energy were calculated with the YASARA plugin FoldX after pKa correction, solvent MD and energy minimization. Calculated and experimental K_i_ (~K_D_) according to ΔG = −RT ln K are given in kJ/mol and as kcal/mol from the FoldX output for comparison. The PRODIGY server was usually run with unchanged PDBs in the protein-protein mode. In case of completely inconsistent results of the calculation the data are shown in brackets. Inhibitor fragment sequences refer to SFTI-1 variants. Discrepancies between calculated and measured data can be explained by the artificial crystallization conditions and the in vitro experiments with varying pH and ionic strength. A better correlation was seen for energy minimized coordinates of 5MCQ.

Complex	FoldX ΔG			K_i_ (exp)	ΔG (exp)	ΔG Prodigy	Structure
Protease/Inhibitor	kJ/mol	kcal/mol	nM	nM	kJ/mol	kJ/mol	PDB
β-Try/PABA	−29.02	−6.93	8220	6100 [25]	−29.79	−23.93 ^1^	3GY4 [26]
Try-3/bikunin-D2	−40.96	−9.79	78	138 [27]	−39.87	−43.10	4U30 [27]
matriptase/SFTI-1	−48.02	−11.47	3.83	0.92 [28]	−51.55	−39.75	3P8F [29]
plasmin/SFTI-Y^4^K^5^R^7^N^14^	−50.41	−12.04	1.46	1.20 [30]	−50.98	−54.03	6D3Z [30]
β-Try/SFTI-T^2^R^5^N^12^N^14^	−48.74	−11.65	2.86	0.70 [15]	−52.23	−48.53	6BVH [15]
KLK4/SFTI-F^2^Q^4^R^5^N^14^	−58.95	−14.09	0.046	0.039 [31]	−59.40	(−41.84)	4KEL [31]
β-Try/SFTI-1	−63.18	−15.10	0.0066	0.017 [32]	−61.47	(−51.46)	1SFI [33]
β-Try/BPTI	−61.42	−14.68	0.017	0.00006 [34]	−75.43	(−51.46)	2PTC [35]
α-thrombin/hirudin-v2	−78.95	−18.87	0.000015	0.000022 [36]	−77.91	(−49.37)	4HTC [37]
legumain/cystatin E	(−31.88)	−7.62	46.4/19.8 ^2^	0.0107 [38]	−62.59/43.95 ^2^	−41.84	4N6O [39]
MPro/cyclo-14-mer	(−30.46)	−7.28	17	14 [40]	−44.81	−44.33	7RNW [40]
BACE-1/22-mer	−53.68	−12.83	0.39/10.0	3.2 ^3^ [41]	−48.46	−45.60/47.28	5MCQ [41]
HIV/cyclo-9-mer	−51.97	−12.42	0.779	4.02 ^3^ [42]	−47.90	(−36.00)	7YF6 [42]
MMP-14/TIMP-2	−52.09	−12.45	0.740/0.149	0.104 [43]	−56.95	−56.07	1BUV [44]
MMP-3/TIMP-1	(−65.90)	−15.75	0.003/0.087 ^4^	0.130 [45]	−56.40	−57.32 ^4^	1UEA [46]

^1^ Protein–ligand mode; ^2^ legumain–cystatin E-K75A; ^3^ IC_50_/2; ^4^ chains A/B with selenomethionines replaced by methionines and two Ala mutations reverted to the native Asn, calculated K_i_ from FoldX/PRODIGY with catalytic Zn^2+^.

## Data Availability

Data contained within the article.
